# The Role of Cell Proliferation and Extracellular Matrix Accumulation Induced by Food Additive Butylated Hydroxytoluene in Uterine Leiomyoma

**DOI:** 10.3390/nu13093074

**Published:** 2021-08-31

**Authors:** Yi-Fen Chiang, Hsin-Yuan Chen, Mohamed Ali, Tzong-Ming Shieh, Yun-Ju Huang, Kai-Lee Wang, Hsin-Yi Chang, Tsui-Chin Huang, Yong-Han Hong, Shih-Min Hsia

**Affiliations:** 1 School of Nutrition and Health Sciences, College of Nutrition, Taipei Medical University, Taipei 11031, Taiwan; yvonne840828@gmail.com (Y.-F.C.); hsin246@gmail.com (H.-Y.C.); yjhunag@stust.edu.tw (Y.-J.H.); 2 Department of Nutrition, I-Shou University, Kaohsiung 84001, Taiwan; yonghan@isu.edu.tw; 3 Clinical Pharmacy Department, Faculty of Pharmacy, Ain Shams University, Cairo 11566, Egypt; mohamed.aboouf@pharma.asu.edu.eg; 4 School of Dentistry, College of Dentistry, China Medical University, Taichung 40402, Taiwan; tmshieh@mail.cmu.edu.tw; 5 Department of Biotechnology and Food Technology, Southern Taiwan University of Science and Technology, Tainan 71005, Taiwan; 6 Department of Nursing, Ching Kuo Institute of Management and Health, Keelung 20301, Taiwan; kellywang@tmu.edu.tw; 7 Graduate Institute of Metabolism and Obesity Sciences, College of Nutrition, Taipei Medical University, Taipei 11031, Taiwan; hsinyi.chang@tmu.edu.tw; 8 Graduate Institute of Cancer Biology and Drug Discovery, College of Medical Science and Technology, Taipei Medical University, Taipei 11031, Taiwan; tsuichin@tmu.edu.tw; 9 School of Food and Safety, Taipei Medical University, Taipei 11031, Taiwan; 10 Nutrition Research Center, Taipei Medical University Hospital, Taipei 11031, Taiwan

**Keywords:** butylated hydroxytoluene, leiomyoma, uterine fibroids, extracellular matrix, matrix metalloproteinase, environmental exposure

## Abstract

Leiomyoma is the most common benign uterine tumor in reproductive-age women. Increasing numbers of studies are focusing on the effects of environmental exposure on the incidence and progression of tumors. One major step taken in the food industry is the addition of food preservatives to maintain freshness. Butylated hydroxytoluene (BHT) is a synthetic phenolic antioxidant, which is widely used as an additive to develop fat-soluble characteristics, as well as in cosmetics and rubber. Previous studies also highlighted that BHT may be related to increased fibrosis capacity and carcinogenic effects. In this study, we explored the effects of the commonly used food additive BHT on leiomyoma progression, and the related mechanism. The exposure of the ELT-3 leiomyoma cell line to BHT for 48 h increased the proliferative effect. Since leiomyoma progression is related to increases in extracellular matrix (ECM) accumulation and matrix metalloproteinase (MMP), BHT could effectively increase ECM-related protein expression, as well as MMP-2 and MMP-9 protein expression. This increase in ECM, in response to BHT, may be linked to the activation of the phosphoinositide 3-kinase (PI3K)/Akt and mitogen-activated protein kinase (MAPK) signaling pathway. Through PI3K inhibition, BHT’s effect on leiomyoma progression could be partially modulated. These results suggest the harmful effect of BHT exposure on leiomyoma progression may relate to PI3K modulation. However, an in vivo study is necessary to confirm these findings.

## 1. Introduction

Leiomyoma (aka uterine fibroids) are the most common benign uterine tumors in reproductive-age women, with an incidence rate of more than 70% [1,2]. Clinically, the leiomyoma is categorized according to its location. The International Federation of Gynecology and Obstetrics (FIGO) classified the definition, submucosal myomas (FIGO type 0, 1, 2) and intramural myomas (FIGO type 3, 4, 5). Submucosal myomas were located below the endometrium, and intramural myomas were located within the uterine wall [3,4]. The main symptoms of leiomyoma are abnormal vaginal bleeding, lower abdominal pain, and bulk symptoms [5]. Recently, studies have reported links between leiomyoma and recurrent miscarriage and infertility [5,6].

Although the pathogenesis of uterine leiomyoma is still not entirely clear, one of the most commonly accepted hypotheses is the accumulation of extracellular matrix (ECM) [7,8]. Extracellular matrix deposition contributes to the amassing of symptoms and the firmness of the tumors, and studies have shown that ECM can enhance the excessive proliferation [9] of the uterine myometrium in a process called mechanotransduction [10]. The components of the ECM include collagen (COL1A1), fibronectin, and proteoglycan.

Leiomyoma cells express significantly higher levels of ECM components than normal uterine smooth muscle cells [11]. Under normal conditions, the ECM is degraded by matrix metalloproteinases (MMPs), of which MMP-2 and MMP-9 are enzymes that mainly degrade collagen [12,13]. In turn, the activity of MMPs is regulated via tissue inhibitors of metalloproteinase (TIMPs) [13,14]; therefore, the balance between MMPs and TIMPs regulates the remodeling of ECM.

With the progression of ECM deposition, intracellular signaling pathways are triggered, such as the mitogen-activated protein kinase (MAPK) and PI3K/Akt (protein kinase B (PKB)) pathways, which increase proliferation and cell survival and maintain the ECM’s deposition microenvironment in leiomyoma [15,16].

Considering the rapidly increasing consumption of food additives, more and more studies are revealing their potentially harmful and toxic effects. To preserve food freshness, antioxidant additives are widely used. Butylated hydroxytoluene (BHT) is one of the most commonly used antioxidant additives, which can improve the stability of fat-soluble vitamins and cosmetics and prevent spoilage [17]. As food antioxidants, the Joint Committee of Experts from FAO/WHO point out the consumption of BHT, its acceptable daily intake (ADI) should not be higher than 0.5 mg/kg body weight [18]. In cosmetics formulations, BHT was used in a wide range, from 0.0002% to 0.5% [19]. In the pulmonary fibrosis animal model, BHT was used as a successful model, with significant endothelial injury and fibrosis phenomenon [20,21]. Additionally, BHT was found to have a systemic effect on the lung, reproductive system, liver, and kidney [17]. However, previous studies have shown that the consumption of BHT could induce lung carcinogenesis [22]. Notably, BHT’s role in leiomyoma is still not clear. The aim and the novelty of this study were to investigate the role of BHT in leiomyoma progression.

## 2. Materials and Methods

### 2.1. Cell Culture and Treatments

The Eker rat-derived uterine leiomyoma ELT3 cell line was provided by Dr. Lin-Hung Wei (Department of Oncology, National Taiwan University Hospital, Taipei, Taiwan). Cells were cultured in Dulbecco’s modified Eagle medium/Ham’s F-12 Medium in a 1:1 ratio (CAISSON Labs, Smithfield, UT, USA), supplemented with 10% fetal bovine serum (FBS; GIBCO, Grand Island, NY, USA), 100 units/mL penicillin (CORNING; Manassas, VA, USA), 100 μg/mL streptomycin, sodium bicarbonate (2.438 g/L, BioShop, Burlington, ON, Canada), and 4-(2-Hydroxyethyl)piperazine-1-ethanesulfonic acid (HEPES; 5.986 g/L; BioShop) under cultured conditions (37 °C, 5% CO_2_) [23]. 

The cells were starved in serum-free medium for 24 hours and then treated with BHT in 1% FBS medium for 24, 48, and 72 h.

### 2.2. Cell Viability Assay

The effect of BHT (Sigma-Aldrich, St. Louis, MO, USA) on cell viability was analyzed using the MTT (3-[4,5-dimethyl-2-thiazolyl]-2,5-diphenyl-2H-tetrazolium bromide; Abcam, Cambridge, MA, USA) assay. After treatments, 1 mg/mL MTT in phosphate-buffered saline was added and incubated for an additional 3 h. The formazan crystals were dissolved in 100 μL dimethyl sulfoxide (DMSO; ECHO Chemical Co. Ltd., Taipei, Taiwan). The optical density was measured using a VERSA Max microplate reader (Molecular Devices, San Jose, CA, USA) at 570 nm and 630 nm. We used the absorbance of the control group as the denominator to calculate the cell viability percentage.

### 2.3. Colony Formation

Cells were seeded in 6-well plates (500 cells/well) and treated with different concentrations of BHT for 48 h. After 48 h, we removed the medium and replaced it with a completed medium, which we then cultured for 1 week. The colonies were fixed with methanol (Echo Chemical Co. Ltd.) and stained with 0.5% crystal violet (Sigma-Aldrich) [24]. We then added DMSO to dissolve the crystal violet and used a VERSA Max microplate reader to measure the absorbance (595 nm). We used the absorbance of the control group as the denominator to calculate the percentage changes.

### 2.4. Immunofluorescence

After the treatments, the cells were fixed in 4% paraformaldehyde (Sigma-Aldrich) for 10 minutes at room temperature, treated with 0.5% Triton X-100 in PBS for 10 minutes, and then blocked with 5% bovine serum albumin (BSA for 30 minutes at room temperature), following with previous study [25]. The cells were then incubated with anti-MMP-2 (1:200, Abcam) or anti-MMP-9 (1:200, Santa Cruz Biotechnology, Santa Cruz, CA, USA) diluted in 5% BSA overnight at 4 °C, followed by Alexa Fluor 448-goat anti-rabbit Immunoglobulin or Alexa Fluor 546-goat anti-mouse Immunoglobulin antibodies (Thermo Fisher Scientific, Waltham, MA, USA) for 1 h at room temperature. Photographs were taken under a fluorescence microscope, then Image J was used to quantify the fluorescence intensity.

### 2.5. Protein Preparation and Western Blot

Cell lysates were homogenized with ice-cold RIPA buffer containing protease (Roche, Basel, Switzerland) and phosphatase inhibitor (Roche, Basel, Switzerland). Following quantification, 30 μg of protein was boiled for 5 minutes, then separated using 10% or 15% SDS–polyacrylamide gel electrophoresis and transferred to polyvinylidene fluoride membranes (0.22 µm). Nonspecific binding sites were blocked with blocking buffer (5% BSA) for 1 h at room temperature, and the membranes were incubated with the primary antibodies for proliferating cell nuclear antigen (PCNA) (1:1000, Cell signaling), matrix metallopeptidase 9 (MMP-9) (1:1000, Santa Cruz Biotechnology), MMP-2 (1:1000, Abcam), collagen type I (COL1A1) (1:1000, Genetex), alpha-smooth muscle actin (α-SMA) (1:1000, Genetex (Irvine, CA, USA), PI3K (1:1000, Cell Signaling Technology, Danvers, MA, USA), p-Akt (1:1000, Cell Signaling), Akt (1:1000, Cell Signaling), extracellular-signal-regulated kinase (ERK) (1:1000, Cell signaling), p-ERK (1:1000, Cell signaling), p38 MAPKinase (1:1000, Cell signaling), p-p38 (1:1000, Cell signaling), and glyceraldehyde-3-phosphate dehydrogenase (GAPDH) (1:10000, Proteintech, Rosemont, IL, USA) at 4 °C overnight. The membranes were washed and incubated for 2 h with anti-rabbit/mouse IgG coupled with alkaline phosphatase (1: 10,000) and then washed with TBST buffer. The bands were detected using ECL and visualized with the eBlot Touch Imager tm (eBlot Photoelectric Technology, Shanghai, China). The values shown were normalized to the internal control GAPDH and analyzed via the ImageJ software. 

### 2.6. Statistical Analysis

Data are expressed as mean ± standard deviation. Statistical analysis was performed with Graphpad Prism version 9 (GraphPad Software, Inc., San Diego, CA, USA), using Student’s *t*-test and one-way analysis of variance (ANOVA), and we used Tukey’s test for post-mortem analysis. *p* < 0.05 indicates a statistically significant difference

## 3. Results

### 3.1. Effects of BHT on Leiomyoma Proliferation

#### 3.1.1. Proliferative Effect

**Figure 1:**
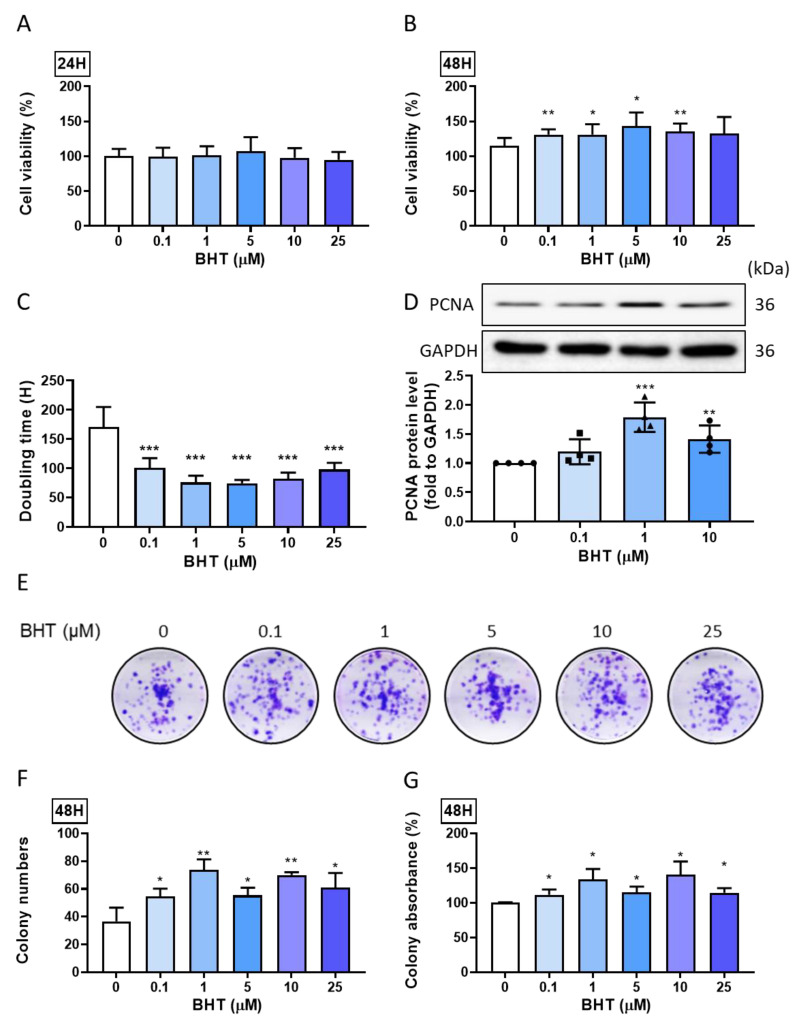
Butylated hydroxytoluene (BHT) effects on leiomyoma proliferation. ELT-3 cells were cultured in a 96-well plate (3000 cells/well) after starvation with a serum-free medium for 24 h. They were then treated with different concentrations of BHT for 24 and 48 h, and we performed the following assays: (**A**) MTT assay to evaluate the cell viability for 24 h and (**B**) 48 h, used doubling time formula to calculate the (**C**) doubling time and Western blot for (**D**) proliferating cell nuclear antigen (PCNA) expression; (**E**) colony formation assay, following culturing in a 6-well plate for the analysis of the long-term effect of different concentrations of BHT; (**F**) graphical representation of colony numbers and (**G**) absorbance percentage following BHT exposure at different concentrations. ImageJ was used to determine the colony number. *, *p* < 0.05; **, *p* < 0.01; and ***, *p* < 0.001, compared with the control group. Doubling time = duration ∗ log (2)/(log (final concentration) −log (initial concentration)).

We used MTT as the cell proliferation assay to evaluate the changes in cell viability following BHT exposure in ELT-3 cells. The ELT-3 cells were seeded in a 96-well plate for 24 h. After starvation for 24 h, they were treated with a graded concentration of BHT (0.1–25 µM) for 24 and 48 h. All the concentrations used for the 48 h treatments could significantly increase leiomyoma cell viability (Figure 1A,B), indicating the potential role of BHT in leiomyoma cell viability. The doubling time of BHT treatments significantly decreased the doubling time (Figure 1C); moreover, the PCNA expression (Figure 1D) would increase after BHT treatments, showing that BHT could increase the ELT-3 cell proliferation. 

#### 3.1.2. Colony Formation

To investigate the long-term effect of BHT on ELT-3 cell proliferation and its ability to stimulate stem cell characteristics, such as colony formation, a colony formation assay was performed after BHT exposure for 48 h and the replacement of the complete medium. The results show that BHT could significantly increase colony formation (Figure 1E), as evidenced by the increased colony count assessed via image J (Figure 1F). Furthermore, we used DMSO to dissolve the staining and measure the absorbance (Figure 1G). Overall, BHT exposure could significantly enhance colony progression and leiomyoma’s proliferation potential. 

### 3.2. Effects of BHT on MMP Modulation

#### 3.2.1. Effects of BHT on MMP-9 and MMP-2 Protein Expression Using Immunofluorescence

**Figure 2:**
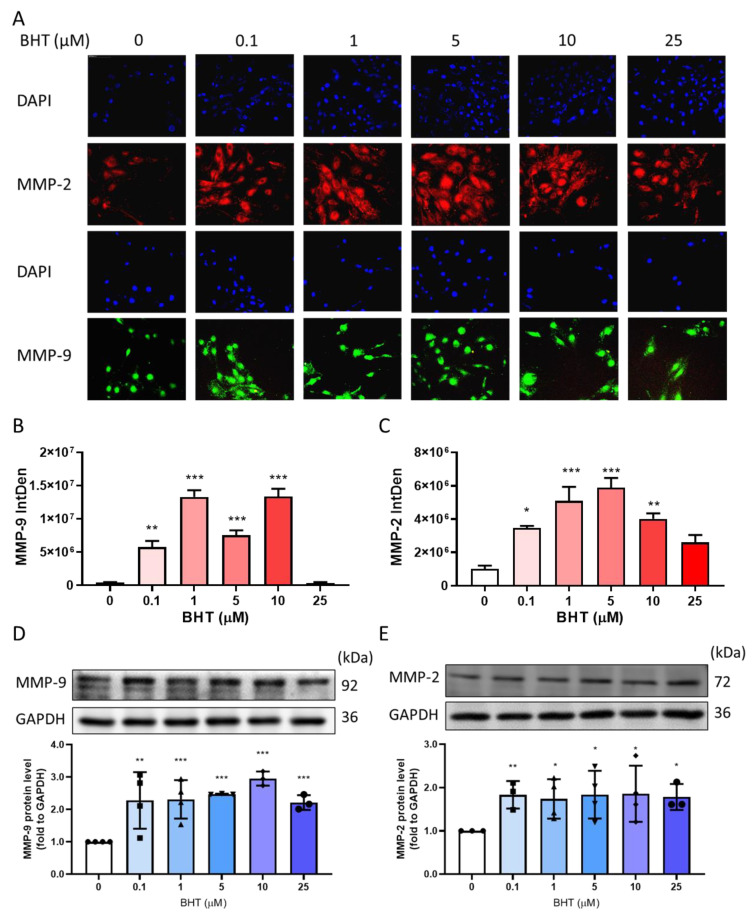
Effects of BHT on MMP modulation. ELT-3 cells were cultured in DMEM/F12 medium. After serum-free starvation for 24 h, cells were treated with different concentrations of BHT for 48 h, and the following experiments were performed: (**A**) immunofluorescence to measure expression changes, employing graphical representations of the fluorescence intensity levels of (**B**) MMP-9 and (**C**) MMP-2. Additionally, increased (**D**) MMP-9 and (**E**) MMP-2 protein expression (*n* = 3–4) are shown. *, *p* < 0.05; **, *p* < 0.01; ***, *p* < 0.001, compared with the control group. We used a fluorescent microscope at 40× magnification. IntDen: integrated density.

Immunofluorescence was used to measure the protein expression of MMP-9 and MMP-2 following 48 h of BHT exposure. The intensity of fluorescence was significantly increased in both MMP-9 and MMP-2, indicating that BHT enhanced the levels in live cells (Figure 2A–C).

#### 3.2.2. Effects of BHT on MMP-9 and MMP-2 Protein Expression Using Western Blot

Matrix metalloproteinases act as a regulator of ECM accumulation. Western blot analysis confirmed that BHT exposure could significantly increase MMP-9 (Figure 2D) and MMP-2 (Figure 2E) protein expression. 

### 3.3. Effect of BHT on Extracellular Matrix Related Proteins

#### 3.3.1. Effects of BHT on ECM Related Protein Expression Using Immunofluorescence

**Figure 3:**
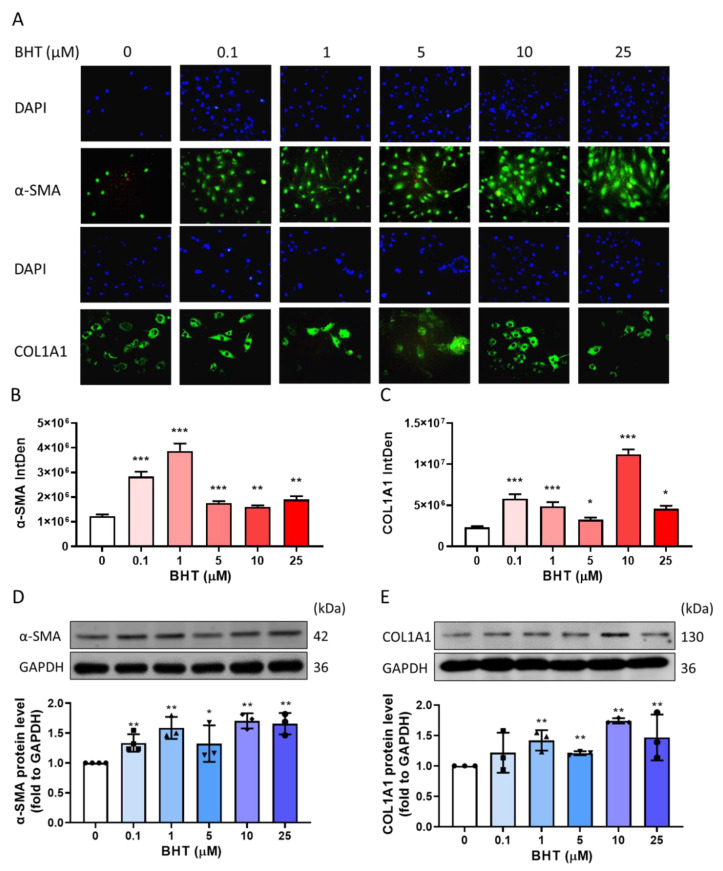
Effect of BHT on extracellular protein expression change. ELT-3 cells were cultured in DMEM/F12 medium. After serum-free starvation for 24 h, cells were treated with different concentrations of BHT for 48 h, and the following experiments were performed: (**A**) immunofluorescence to measure expression changes, employing graphical representations of the fluorescence intensity levels of (**B**) α-SMA and (**C**) COL1A1. Additionally, increased (**D**) α-SMA and (**E**) COL1A1 protein expression (*n* = 3–4) are shown. *, *p* < 0.05; **, *p* < 0.01; ***, *p* < 0.001, compared with the control group. We used a fluorescent microscope at 40× magnification. IntDen: integrated density.

Immunofluorescence was used to measure the protein expression of α-SMA and COL1A1 following 48 h of BHT treatment. The intensity of fluorescence was significantly increased in both α-SMA and COL1A1 (Figure 3A–C).

#### 3.3.2. Effects of BHT on ECM Related Protein Expression Using Western Blot

The overexpression of extracellular matrix-related proteins contributes to leiomyoma progression. Therefore, we explored ECM-related protein expression in response to BHT treatments, including COL1A1 and α-SMA as confirmation for previous immunofluorescence results. Our results were consistent with the fluorescence intensity results. Moreover, BHT exposure induced the significant protein expression of COL1A1 and α-SMA (Figure 3D,E). Collectively, these results indicate that the BHT exposure could enhance ECM accumulation in leiomyoma.

### 3.4. PI3K/Akt and MAPK Signaling Related Protein Expression Change in BHT Induced ECM Accumulation

**Figure 4:**
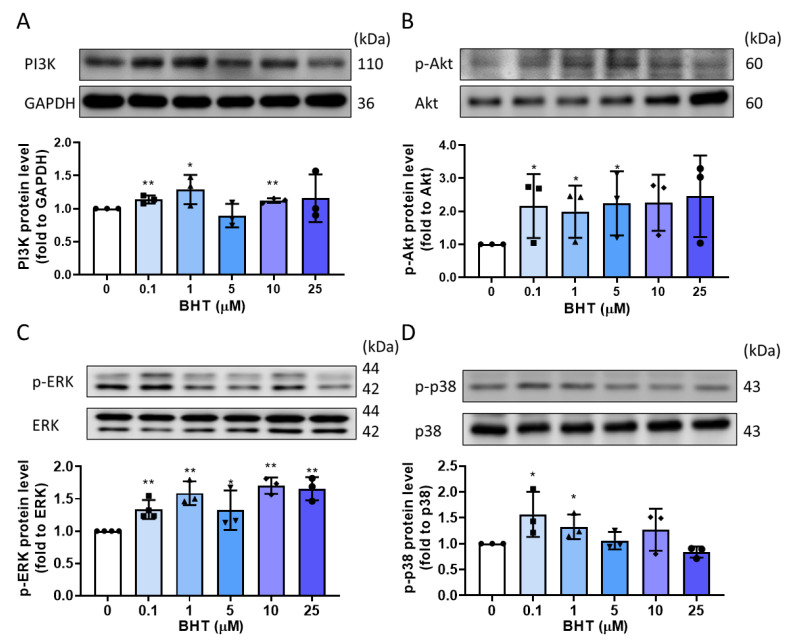
The PI3K/Akt and MAPK signaling pathway involved in BHT-induced ECM accumulation. ELT-3 cells were cultured in DMEM/F12, followed by serum-free starvation for 24 h. Cells were treated with different concentrations of BHT for 48 h. Western blotting was used to explore the protein expression of (**A**) PI3K (**B**) p-Akt/Akt (**C**) p-ERK/ERK, and (**D**) p-p38/p38 protein expression (*n* = 3). *, *p* < 0.05; **, *p* < 0.01; and ***, *p* < 0.001 compared with the control group. GAPDH was used as loading control.

One of the well-known triggering factors that regulate extracellular–intracellular signaling is ECM [15]. Studies showed that activation of PI3K/Akt and MAPK signaling pathways could modulate ECM progression [26]. Therefore, we sought to explore whether BHT mediated ECM induction is accompanied by activation of the PI3K/Akt pathway. ELT-3 cells exposure with BHT for 48 h resulted in an increase in PI3K and *p*-Akt/Akt protein expression (Figure 4A,B), and in low doses, BHT could activate MAPK signaling transduction (Figure 4C,D), indicating that BHT exposure could activate PI3K/Akt and MAPK signaling pathway to increase the ECM accumulation. 

### 3.5. The Potential Modulative Signaling Pathway in BHT Induced ECM Accumulation

**Figure 5:**
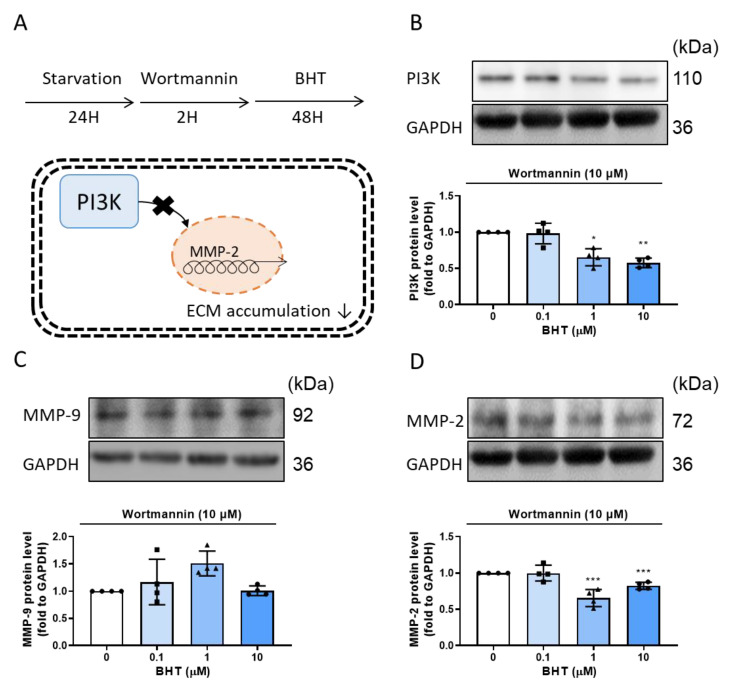
The potential modulative signaling pathway in BHT0induced ECM accumulation: (**A**) the flowchart shows that treated with PI3K inhibitor wortmannin for 2 h and treated with BHT for 48 h to evaluate the PI3K modulated BHT’s effect in ECM accumulation. After treatment, Western blot analysis was used to evaluate the (**B**) PI3K, (**C**) MMP-9, and (**D**) MMP-2 protein expression. *, *p* < 0.05; **, *p* < 0.01; and ***, *p* < 0.001 compared with the control group. GAPDH was used as loading control.

PI3K inhibitor, wortmannin, was used to investigate the potential modulator of BHT on ECM accumulation. According to a previous study, PI3K acts as the important modulator of MMP-2 [27]. By using wortmannin, the results indicated that the PI3K inhibition could reverse the BHT’s effect on ECM accumulation (Figure 5).

## 4. Discussion

In this study, BHT exposure showed its ability on the progression of uterine leiomyoma by increasing the proliferation and extracellular matrix accumulation effect through PI3K/Akt and MAPK signaling modulation. 

The extracellular matrix is engaged in a complex interaction with the surrounding microenvironment while providing structural support to the cell and tissue [11]. Moreover, ECM could induce signaling networks, which, in turn, induce further ECM synthesis and deposition [11]. In leiomyoma, ECM turnover and remodeling are disrupted, with the overexpression of related proteins, such as collagen, fibronectin, and α-SMA [7]. The key modulator of ECM accumulation is the matrix metalloproteinase, which participates in tissue remodeling and modulated the tissue inhibitor of metalloproteinases (TIMPs) in leiomyoma [28]. Leiomyoma pathogenesis involves growth factor stimulation, with subsequent cell proliferation, inflammation, and fibrosis [29]. Several signaling pathways are involved in leiomyoma progression, including the MAPK signaling pathway, the phosphorylation of extracellular signal-regulated kinases (ERK), the phosphoinositide 3-kinase (PI3K)/Akt pathway, and the wingless-type (Wnt)/β-catenin signaling pathway [30].

The PI3K/Akt pathway participates in the regulation of mammalian target of rapamycin (mTOR), and modulates cancer cell survival, proliferation, and apoptosis, therefore playing an important role in cancer progression [24] and in leiomyoma [31].

The pathogenesis of uterine leiomyoma is still not clear, but leiomyoma growth is estrogen- and progesterone dependent, which indicated hormone dependence plays an important role in leiomyoma progression [32,33]. Studies have shown that early exposure to environmental endocrine-disrupting chemicals (EDCs) increases the risk of leiomyoma development later in life [32,34,35]. EDCs are found in sweeteners, preserved food, and food additives [36]. EDCs impart steroidogenesis [37], estrogen-like effects, and promote the development of gynecological tumors.

Food additives maintain quality, taste, and freshness [38]. For food spoilage prevention, these food antioxidant agents are one of the major classes of food additives [39]. The most commonly used category is synthetic phenolic antioxidants, such as butylated hydroxytoluene (BHT) and butylated hydroxyanisole (BHA). These food additives were approved by the US Food and Drug Administration. However, their side effects for humans are still being debated; for example, their consumption increases oxidative stress, carcinogenicity, reproductive toxicity, and DNA repair defects [40,41]. Therefore, BHT has been restricted in some food additives, but females can still be exposed via cosmetics and medicine consumption [19], in addition to exposure to rubber, plastics, and even the environment [42], with long-term effects.

Studies exploring the toxicity of BHT have been inconsistent. One study highlighted its positive effects based on its antioxidant activity, ability to increase intracellular antioxidant enzyme levels [43], and anti-cancer effects [19], while several other studies have indicated that BHT may induce kidney and liver damage [19]. Notably, BHT metabolites are related to toxicity, as shown in a study that used gas chromatography coupled to mass spectrometry (GC-MS) [44]. Based on BHT’s structure, it has a greater ability to accumulate in adipose tissue and affect hormone regulation in mammary glands, as well as being transferred through the placenta [45].

BHT was found to be harmful to metabolic- and reproductive-related diseases. In reproductive disorders, the exposure of mouse Leydig cells (TM3) with BHT suppressed cellular proliferation, altered the cell cycle, and changed the cytosolic and mitochondria calcium homeostasis [46]. In addition to causing endoplasmic reticulum (ER) stress and increasing DNA damage, it further triggered the apoptosis signaling pathway, which, in turn, activated the PI3K/Akt and MAPK signaling pathways, eventually promoting carcinogenesis [46].

Antioxidant enzymes could eliminate oxidative stress. Manganese superoxide dismutase (MnSOD), on the other hand, could act as a tumor suppressor [47] and promotor [48]. MnSOD, a highly antioxidative compound, promoted metastatic effects through the upregulation of MMP-2 [49]. In lung cancer patients, MnSOD-positive tumors were related to higher MMP-2 expression and caused tumorigenesis, including proliferation and fibrosis progression [50]. Anti-oxidation or oxidative stress would regulate MMP activation [51]. High doses of MnSOD, with its ability to eliminate oxidative stress, had a tumor-suppressor effect in several cancers [52], while low doses caused no changes in oxidative stress, leading to the accumulation of reactive oxygen species (ROS) and stimulating cancer progression through increased MMP activity [49]. Incomplete ROS degradation may trigger MMP-2 activation [53], and therefore, the different effects of the antioxidant and the additive antioxidant should be considered. Our results also revealed that BHTs’ effect on ECM-related protein expression may vary with different dosages—in low dosage exposures, they are most effective.

Studies showed that BHT interacts with PI3K/Akt and MAPK signaling modulation and enhances fibrosis [54]. An injection of 400 mg/kg BHT in BALB/C mice resulted in significant intestinal fibrosis and lung fibrosis within 14 days [55]. Additionally, BHT could induce lung carcinogens and lung damage, as shown in transgenic mice with the rasH2 gene that underwent exposure with BHT for 9 weeks [56]. BHT exposure also increased collagen I, III, and V expressions and altered both the telomerase and apoptosis-related expressions, along with further epithelial cell injury. Collectively, these studies indicate that BHT potentially affects fibrosis progression [57]. 

In the current study, BHT’s role in leiomyoma progression was explored for the first time. BHT increased proliferative effect, increased ECM-related protein expression, and induced ECM accumulation, which is essential to leiomyoma progression. Additionally, in vitro experiments showed that BHT exposure could alter protein expression, in addition to activating the PI3K/Akt and MAPK signaling pathway. The study shows, for the first time, that BHT exposure could increase the leiomyoma progression, indicating that it may participate in PI3K and MAPK signaling pathways. However, the in vitro study used could not fully explain the role of BHT in leiomyoma. The metabolites of BHT may play different roles in leiomyoma progression; therefore, further animal studies are needed to realize its effect in the future.

## 5. Conclusions

**Figure 6:**
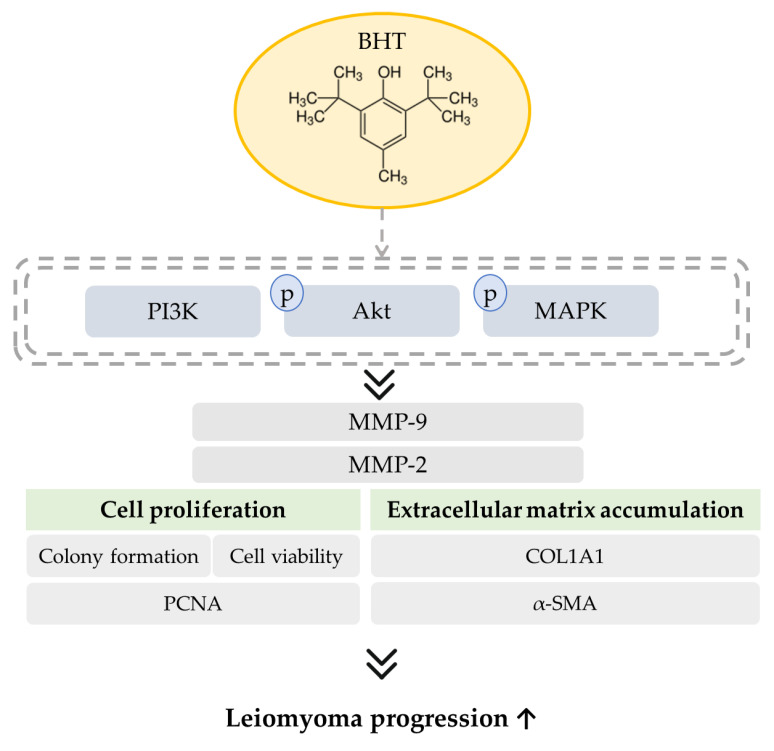
Schematic representation of the potential effects of BHT in leiomyoma progression. BHT could increase cell proliferative effect, modulate PI3K/Akt and MAPK signaling pathways, increase MMP enzyme and protein expression, and increase the ECM-related protein expression.

Environmental exposure to BHT could be associated with several disease disorders and disadvantages. Using the ELT-3 rat leiomyoma cell model, the results shed light on BHT’s potential in enhancing leiomyoma cell proliferation, colony formation, and ECM accumulation in a mechanism that might involve modulation of PI3K/Akt and MAPK signaling pathways (Figure 6). It is the first study to explore the effect of food additives on leiomyoma progression. Further studies will be needed in the future to investigate the pro-fibroid effects of BHT metabolites in animal studies.

## Data Availability

The data presented in this study are available on request from the corresponding author.

## Abbreviations

α-SMA	Alpha smooth muscle actin
BHA	Butylated hydroxyanisole
BHT	Butylated hydroxytoluene
BSA	Bovine serum albumin
COL1A1	Collagen, type I
ECM	Extracellular matrix
EDCs	Environmental endocrine-disrupting chemicals
h	Hours
MAPK	Mitogen-activated protein kinase
MMP	Matrix metalloproteinase
MnSOD	Manganese superoxide dismutase
MTT	(3-(4,5-dimethylthiazol-2-yl)-2,5-diphenyltetrazolium bromide)
PCNA	Proliferating cell nuclear antigen
PI3K	Phosphoinositide 3-kinase
PKB	Protein kinase B
ROS	Reactive oxygen species
TIMPs	Tissue inhibitor of metalloproteinase
